# Institutional capacity for health systems research in East and Central Africa schools of public health: enhancing capacity to design and implement teaching programs

**DOI:** 10.1186/1478-4505-12-22

**Published:** 2014-06-02

**Authors:** Mabel N Nangami, Lawrence Rugema, Bosena Tebeje, Aggrey Mukose

**Affiliations:** 1 Department of Health Policy and Management, College of Health Sciences, School of Public Health, Moi University, P.O. Box 4606, Eldoret 30100, Kenya; 2 Department of Community Health, School of Public Health, National University of Rwanda, P.O. Box 5229, Kigali, Rwanda; 3 Maternal and Reproductive Health, College of Public Health and Medical Sciences, Jimma University, P.O Box 378, Jimma, Ethiopia; 4 Department Epidemiology and Biostatistics, College of Health Sciences, School of Public Health, Makerere University, P.O Box 7072, Kampala, Uganda

**Keywords:** Curriculum design, Health systems research, Teaching health systems research, Training in health systems research

## Abstract

**Background:**

The role of health systems research (HSR) in informing and guiding national programs and policies has been increasingly recognized. Yet, many universities in sub-Saharan African countries have relatively limited capacity to teach HSR. Seven schools of public health (SPHs) in East and Central Africa undertook an HSR institutional capacity assessment, which included a review of current HSR teaching programs. This study determines the extent to which SPHs are engaged in teaching HSR-relevant courses and assessing their capacities to effectively design and implement HSR curricula whose graduates are equipped to address HSR needs while helping to strengthen public health policy.

**Methods:**

This study used a cross-sectional study design employing both quantitative and qualitative approaches. An organizational profile tool was administered to senior staff across the seven SPHs to assess existing teaching programs. A self-assessment tool included nine questions relevant to teaching capacity for HSR curricula. The analysis triangulates the data, with reflections on the responses from within and across the seven SPHs. Proportions and average of values from the Likert scale are compared to determine strengths and weaknesses, while themes relevant to the objectives are identified and clustered to elicit in-depth interpretation.

**Results:**

None of the SPHs offer an HSR-specific degree program; however, all seven offer courses in the Master of Public Health (MPH) degree that are relevant to HSR. The general MPH curricula partially embrace principles of competency-based education. Different strengths in curricula design and staff interest in HSR at each SPH were exhibited but a number of common constraints were identified, including out-of-date curricula, face-to-face delivery approaches, inadequate staff competencies, and limited access to materials. Opportunities to align health system priorities to teaching programs include existing networks.

**Conclusions:**

Each SPH has key strengths that can be leveraged to design and implement HSR teaching curricula. We propose networking for standardizing HSR curricula competencies, institutionalizing sharing of teaching resources, creating an HSR eLearning platform to expand access, regularly reviewing HSR teaching content to infuse competency-based approaches, and strengthening staff capacity to deliver such curricula.

## Introduction

A number of studies recognize the central role that a competent health workforce plays in achieving the Millennium Development Goals and Universal Health Coverage in sub-Saharan Africa [1-4]. Health systems research (HSR) is recognized as a core component of a functioning and responsive health system. However, health workforce studies rarely assess the capacity of training institutions to produce competent research practitioners who are able to function effectively in inter-professional teams [5]. The *Lancet*’s seminal publication on transforming health professional education for the 21st century [5] and the recent study by the Consortium for Health Policy and Systems and Analysis in Africa (CHEPSAA) on the capacity of seven African universities to teach HSR [6] reveal three key issues: (i) a piecemeal shift from traditional education models; (ii) a lack of clarity on competencies for HSR graduates and content of curriculum; and (iii) less focus on the training (teaching) process as opposed to training output.

On the first challenge of slow adoption to change, Frenk et al. describe a three-generational paradigm shift in models for educational reforms as follows: from a science-based model at the beginning of the 20th century (scientific-based curriculum and traditional teaching methods), to the problem-based model in the mid-20th centuary (problem-based learning and instructional innovations), and propose a systems-based model for the 21st century (competency-driven curriculum and adult learning principles) [5]. The main attraction of the systems-based model is its focus on two outcomes; namely, designing and teaching a competency-driven curriculum that leads to transformative learning and championing institutional reforms that promote interdependence in education. Furthermore, most of the principles of a competency-based education are aligned to principles of health systems. For example, systems approach, people-centered, performance-based, interdependence, integration, team approach, and innovation to training by using technology and adult learning approaches [1,5]. Despite the strong rationale and push for educational reforms, for most African institutions there has been little strategic effort yielding a slow and disjointed process [4].

The next challenge is a lack of clarity as to the nature of the training and competencies of professionals who engage in HSR. The seminal review of over 2,460 medical schools indicated that the curricula are fragmented, redundant, and rarely revised, leading to graduates who are ill-prepared to understand and address the dynamics in the health system [5]. Yet, a competent workforce must exemplify the knowledge, skills, and attitudes that enable the professional to perform their tasks successfully [6,7]. Additionally, there is a lack of agreement between government and external stakeholders and universities on the framework for strengthening the contribution of universities to development in Africa [8,9]. This lack of clarity and appreciation for building capacity for training that is transformative has promoted a culture of project-based training and consulting which encouraged faculty to work in silos rather than teams, and to develop strategic partnerships and networks that promote institutional capacity building in both research and teaching [9].

Finally, although there is an increasing awareness, especially at government level, on the importance of staff development, the emphasis is still on capacity building to conduct research as opposed to capacity building to teach [8,10]. Rarely is the focus on the educators: the teachers or the facilitators. Global efforts to design curricula and train trainers of trainers in HSR started in the early 1980s and, according to Varkevisser et al. [11], key reasons for success of the Joint HSR Project for the Southern African Region were enthusiasm among trainers and trainees and flexibility of the training. The challenges include limited consultation of stakeholders in curricula development, inadequate framework for monitoring the curricula, inadequacy of funding for the projects, high turnover of in-service trainees [11], and the paradigm shift in medical education [12]. Worsening quality of university education is, in part, driven by the exponential expansion in access to university education between 2000 and 2010, unmatched by either a freeze in hiring or a geometric expansion of academic staff [8,10].

At the regional level, the Inter University Council of East Africa (IUCEA) has the mandate to attain and maintain high academic standards through quality assurance in design of competency-based curricula, exchange of students, faculty and external examiners, and collaborative research [13]. Working with the National University Commissions (Kenya and Tanzania) and Councils (Uganda, Rwanda, and Burundi), IUCEA has taken the curriculum as an instrument of quality improvement and developed guidelines for quality assurance framework for academic programs, although these are not yet fully implemented. The IUCEA also has a mandate to promote university involvement in the community by promoting research networks and centers of excellence in research. Quality research is largely dependent on the design and implementation strategies of the relevant academic curricula [4-7,11-22]. The Medical Education Partnership Initiative and CHEPSAA are among the few regional initiatives that attempt to forge a link between design and implementation of a curriculum and competencies of the graduate and health professional.

Perceptions from internal (within institution) and external (external to institution) stakeholders can be sought to inform the nature of the training process as well as relevance and alignment of curricula design to national priorities. This paper reports on an organizational capacity assessment conducted by seven schools of public health (SPHs) from universities in East and Central Africa that sought to explore these very aspects. Jimma University College of Public Health and Medical Science (CPHMS, Ethiopia), Kinshasa School of Public Health (KSPH, Democratic Republic of the Congo (DRC)), Makerere University School of Public Health (MakSPH, Uganda), Moi University School of Public Health (MUSOPH, Kenya), Muhimbili School of Public Health and Social Sciences (MUSPHSS, Tanzania), National University of Rwanda School of Public Health (NURSPH, Rwanda), and University of Nairobi School of Public Health (SPHUoN, Kenya), are collaborating under the Higher Education for Leadership Through Health (HEALTH) Alliance since 2008 [15]. The countries hosting these institutions share similar health system characteristics, which may vary slightly in level and magnitude, include high disease burden, dilapidated health infrastructure, weak leadership, poor management, and inadequate human resources in both numbers and competencies [4-6]. With funding from the Future Health Systems consortium, these seven SPHs established the Africa Hub in 2011 to build capacity for HSR as a means to strengthen the health systems in their respective countries and the region. This is in recognition of the challenges that exist to strengthening local, regional, and national health systems, including a lack of in-country capacity to commission, conduct, and use HSR [1,2,16]. This, in turn, is partly driven by a limited capacity to teach HSR. Most support for research funding tends to focus on providing capacity training for individual faculty rather than an inter-professional team approach. Most funding for HSR is pegged to large research grants with a small component, if any, being earmarked for capacity development for teaching and for research.

It is against this background that the SPHs conducted a multi-site collaborative study to explore the institutional capacity of the SPHs to conduct HSR, conduct knowledge management, teach HSR, and network with national and regional stakeholders in HSR. This paper, one in a series of four [16-18], explores the capacity of SPHs to design and teach HSR curricula. Specifically, it reports on four issues: context for designing and teaching HSR relevant curricula; alignment of the existing curricula design to competency-based principles; perceived interest and capacity of staff to teach HSR curricula; and opportunities for aligning health system priorities to HSR teaching programs.

## Methods

### Study design

The study employed a cross-sectional design, combined both quantitative and qualitative approaches, and used rapid appraisal techniques for an assessment of participant views and perceptions on HSR strengths, weaknesses, and priorities.

### Study population

The study population can be grouped into three main categories: teaching and non-teaching staff at each SPH (internal) who were currently or potentially interested in HSR; external (to SPH) stakeholders within the university who are part of senior management at the university and hold positions relevant to teaching and research; and external (to University) stakeholders within the country who represent industry and academic and research institutions partnering with the host SPH.

### Sampling approach

In each of the seven SPHs, focal persons (FPs) and their teams put together a sampling frame for eligible respondents under each of the three categories. All eligible respondents were purposively sampled and FPs made several attempts to secure completed interviews. The sampled respondents are described under the various tools.

### Data collection

A co-created self-assessment tool was disseminated to all teaching staff across the seven SPHs for their perceptions of HSR capacity at the organizational, not individual, level. Furthermore, a core team from each SPH conducted key informant interviews (KIIs) of internal and external stakeholders that were led by staff from the SPHs; further details are reported elsewhere [18]. This approach was proposed because the intention of the assessment exercise was primarily to provide a systematic method for each of the SPHs to reflect on its strengths and weaknesses with respect to HSR and to stimulate discussion on what kind of strategies would be most effective to help develop HSR capacity, recognizing the country- and school-specific contexts.

This study extracted data relevant to curriculum design and teaching from primary tools for data collection.

•**Individual self-administered tool.** Teaching and non-teaching staff at each SPH were requested to reflect on strengths and weaknesses of their own school with respect to HSR. This tool consisted largely of statements about the school’s capacity, and respondents used a 5-point Likert scale (1 = strongly disagree, 5 = strongly agree) to indicate strength of agreement. Nine questions under the organizational capacity were relevant to course design, teaching, and learning resources.

•**Profile of HSR within the institution.** Data from this tool was gathered from heads of departments, Dean of the SPH, deputy vice-Chancellors in charge of academics, and research and collaborations. This was a form to collect quantitative data about the number of staff working on HSR, the type and diversity of skills represented, and the type of research conducted. Qualitative data collected included a review of whether the content and structure of the different courses taught at each of the seven SPHs was relevant to HSR.

•**A “quick and dirty” exercise** to consult with key in-country stakeholders and researchers at each SPH using a series of semi-structured interviews. The key stakeholders included representatives of Ministries of Health, public health associations, members of parliament, and funders such as WHO, Center for Disease Control and Prevention, Belgian Technical Cooperation, and Jhpiego. Notes were taken during the interviews, but the interviews were not transcribed or formally coded and analyzed.

•**Consultative meetings with faculty** occurred to discuss the findings from the three prior steps.

•Upon completion of document analysis of the assessment results, supplementary data on history of the SPHs and structure of curricula. This was in recognition that although capacity assessment tools explored the number and type of HSR relevant courses being offered, it did not explore the history, philosophy, and the actual content and structure of the said HSR courses. The supplementary data collection was intended to assess the alignment, if any, to competency-based models for curricula and teaching. Each Dean designated a staff member as FP to coordinate all HSR activities. Each SPH was requested to complete a template covering the aforementioned data.

### Overall approach to data management

To ensure reliability of data, the seven participating SPHs adopted various strategies. First, the SPHs implemented a common approach to the protocol based on modifications of IDRC tools developed for organizational capacity assessment [23] and tools for self-assessment and KIIs. In particular, the FPs participated in joint questionnaire design in Kampala in June 2011 and were primarily responsible for piloting the tools, incorporating any changes, collecting and analyzing data, organizing internal result-sharing workshops, documenting capacity development plans, and disseminating findings at a regional workshop held in December 2011 in Nairobi.

Second, as part of the approach, a common definition of HSR was adopted and the information along with examples of what HSR is and is not was inserted at the start of each questionnaire. A common understanding of HSR not only facilitated comparison of findings across the participating SPHs, but also improved consistency in approach to the assessment of capacity for HSR, HSR priorities, and the policy environment that exists at each school.

Finally, during the collection of supplementary data, following the capacity self-assessment, a common reference point of October 2011 was emphasized so as to restrict data to this period.

### Data analysis

The analysis involved triangulation of various data sources, including document reviews, self-assessments, in-depth interviews with key informants, and supplementary information.

### Quantitative data

Of the 26 questions on the self-assessment tools, nine questions were relevant to HSR teaching and curricula design. For these nine questions, average scores calculated for each question, as described elsewhere [18], were extracted for the seven SPHs. For this paper, the proportion of respondents who agreed (score 4) or strongly agreed (score 5) as well as mean scores of values from the 5-point Likert scale (1 = strongly disagree, 5 = strongly agree), were categorized and interpreted to determine the strength of agreement with the relevant statements: very strong (≥3.8), average (3.0–3.79), and very weak (<3.0). The focus of the analysis was to explore the strengths, weaknesses, and challenges of each SPH with respect to these nine elements. Further analysis involved assessment of institutional capacity based on comparison of these strengths and weakness across all SPHs.

On design of HSR-relevant curricula and teaching, the analysis and interpretation are made based on key elements of the framework for competency-based curricula proposed by Frenk et al. [4], which promotes inter- and trans-professional (multidisciplinary admission criteria), is tailored to suit identified competencies, favors continuous rather than summative assessment, is student centered, promotes experiential learning through field visits and practicums, emphasizes an interprofessional/team approach to training, supports adult learning strategies, is community-based, and harnesses the power of innovation using technology. These were interpreted as coalesced information from secondary data review of relevant policy documents, curricula, and the self-assessment tool to determine the extent to which the Masters in Public Health (MPH) curricula are anchored in competency-based principles.

### Qualitative data

In this study, we use two sets of qualitative data to describe views of internal (institutional profile tool) and external stakeholders (unstructured guide) relevant to curriculum design and teaching and health system priorities. The first set includes qualitative statements by key informants in the SPHs and senior management at college and university levels who responded to the institutional profile tool. Relevant themes were identified from these statements and clustered around the framework for competency-based education. Relevant quotes are used to provide explanations on identified strengths or weaknesses with regard to context of courses, design, and capacity to teach HSR.

For qualitative data from the interview guide for external stakeholders, recurring themes on health systems priorities were first collated by each FP. In this study, these themes were clustered and then aligned to the six building blocks taken from the WHO ‘Framework for Action’ [1]. The lists of priority themes from each SPH were used to draw comparisons to determine if there were areas of convergence on health system priorities and curricula offered at the seven SPHs.

FPs organized two stakeholder meetings with these aims. The first was used to build consensus around key issues and themes from the capacity assessment. The second workshop was used to validate the findings and final report.

### Ethical considerations

Ethical approval to conduct this study was granted by the various Institutional Research Boards in member institutions (one exception to this was at MUSPHSS, Tanzania, where the assessment was regarded as part of an ongoing routine capacity strengthening effort). Additionally, each Dean provided a letter of introduction to the research team members. Written informed consent was voluntarily obtained from all respondents. To assure confidentiality, names of study respondents were omitted from the study tools as well as in the analysis and dissemination of the findings.

### Limitations

Limited data was available through the capacity assessment to investigate the relationships between what the curricula intended, what was actually delivered, and the impact. An extended in-depth review would be required to permit a more detailed analysis. The tools and small sample size guide us to focus on determining varying perspectives of internal and external stakeholders to the SPHs without the benefit of interrogating associations and relationships among variables of interest. The qualitative tool for self-assessment was adopted from other studies on institutional capacity assessment [18]. The tool, which covered processes, context, perceptions, and limited outputs, did not, however, collect views from graduates on the impact of curricula.

## Results

A total of 123 faculty and staff completed the self-ssessments and 73 stakeholders were interviewed. Table 1 summarizes the distribution of respondents by institutions.

**Table 1:** Number of respondents by school

**Institution**	**Number of faculty and staff involved in self-assessment**^**1**^	**Number of external stakeholder interviews**
CPHMS, Ethiopia	26	6
KSPH, DRC	35	26
MakSPH, Uganda	15	6
MUSOPH, Kenya	22	15
MUSPHSS, Tanzania	16	4
SPHUoN, Kenya	5	12
NURSPH, Rwanda	4	4
Total	123	73

^1^Respondents to the self-assessment tool includes some of the key informants.

Quantitative and qualitative findings from the self-assessment, institutional profiles, and semi-structured interviews with internal and external stakeholders are presented under the following themes: context of teaching programs in the SPHs; design of existing curricula; perceived interest and competencies to teach, implement, and review HSR curricula; and opportunities for alignment of HSR priorities and teaching programs.

### Context for designing and teaching HSR-relevant programs in the SPHs

Despite the fact that the schools or faculties of medicine were established as early as 1924 (Makerere University), the first institute of public health in the region was not established until the early 1970s. This started with Makerere University in 1974, followed by the University of Kinshasa in 1985. In the 1990s, institutes of public health were established at Muhimbili University (1991) and Moi University (1998), which later became SPHs. More recently, the National University of Rwanda (2000), Jimma University (2009), and the University of Nairobi (2010) established their SPHs. Jimma University and Moi University were founded on the innovative problem-based and community-oriented educational philosophy; the other five schools, through development of new curricula, have evolved and adopted (to varying degrees) the problem-based model of education but none to date have fully embraced competency-based education philosophy.

As shown in Table 2, there are variations in the number and type of departments housed by the respective SPHs. All except CPHMS, Ethiopia, and MUSOPH, Kenya, have the traditional epidemiology and biostatistics department. Similarly, all except MUSPHSS, Tanzania, have a Department of Health Policy and Management, where HSR-relevant training, research, and services are located. All SPHs offer various undergraduate courses relevant to HSR in their respective colleges of health sciences, but only MUSOPH, Kenya, offers an undergraduate degree program in public health. A MPH program is offered by every SPH and each hosts at least one stand-alone short course relevant to HSR. Table 2 shows the variety of Masters programs offered at the SPHs. These include MPH and Masters of Science programs offered in various specializations.

**Table 2:** Profile of the seven schools of public health in East and Central Africa

**University domain**	**CPHMS, Ethiopia**	**KSPH, DRC**	**MakSPH, Uganda**	**MUSPHSS, Tanzania**	**MUSOPH, Kenya**	**SPHUoN, Kenya**	**NURSPH, Rwanda**
Year of establishment of SPH	1983 – as Jimma Institute of Health Sciences and later as Jimma University in 1999 and CPHMS in 2009	1985 –Kinshasa School of Public Health (KSPH)	2008 – as Institute of Public Health and later as School of Public Health	Initially 1991 as Institute of Public Health and in 2003 as School of Public Health and Social Sciences (SPHSS)	1998 – as Institute of Public Health and 2004 as School of Public Health	2010 – School of Public Health, University of Nairobi was established in September 2010 through the transformation of the Department of Community Health	2000 – The National University of Rwanda, School of Public Health (NURSPH) in Butare and moved to Kigali in 2005
Departments in SPH	Health services management; Epidemiology; Population and family health; Health education and behavioral sciences	Public health policy and management; Epidemiology and biostatics; Nutrition; Community health; and Environmental sciences	Health policy, planning, and management; Community health and behavioral sciences; Disease control and environmental health; Epidemiology and biostatistics; Regional Centre for Quality Health Care	Behavioral sciences; Community health; Development studies; Epidemiology and biostatistics; Parasitology and medical entomology; Environmental and occupational health	Health policy and management; Environmental health; Epidemiology and human nutrition	Health care systems and policy development; Epidemiology and biostatistics; Disease prevention, control, and health promotion; Community health sciences	Health policy, economics and management; Epidemiology and biostatistics; Community health. COE in HSS
Undergraduate programs	No programs but offer courses	No programs but offer courses	No programs but offer courses	No programs but offer courses	Environmental health	No programs but offer courses	No programs but offer courses
Postgraduate programs	5 MPH and 1 MSc	1 MPH	3 MPH full time, distance, MPH nutrition and 1 MSc in Health services research	5 MPH and MSc	5 MPH specializations	1 MPH	3 MPH and MSc
Short courses	1	2	9	1	2	2	4

**Figure 1:**
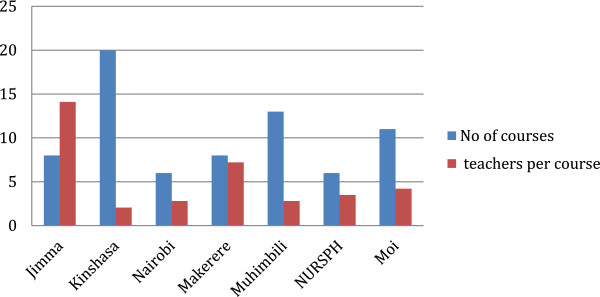
Ratio of full time staff to courses offered across the SPH.

The HSR-relevant courses taught within the MPH programs include health economics, health policy, research methods, epidemiology, and biostatistics. Only two schools offer full-fledged graduate programs that are specific to HSR: Master of Health Services Research (MakSPH, Uganda) and Master of Health Systems Management (KSPH, DRC). As of October 2011, only NURSPH, Rwanda, and MakSPH, Uganda, have received technical support from agencies such as Rockefeller to develop health systems management modules/courses.Figure 1 shows the general course load across the different SPHs. It compares the number of courses and the number of full-time staff at each SPH. This is not a demonstration of the correlation but rather a qualitative assessment of sheer numbers in relation to teaching programs. It does not take into account the research load or engagement for each member of staff. For example, CPHMS, Ethiopia, and MakSPH, Uganda, have the same number of courses (seven) but differ in staffing levels, 14 and 6, respectively.

### Design of existing curricula

Table 3 outlines the structure of the MPH degree program highlighting the similarities and differences among the seven SPHs. The variables, duration, mode of delivery, credit transfer, duration of practicum, admission requirements, assessment format, admission and graduation rates, full time staff, and last curricular review will serve as a reference point for discussions on the structure of teaching programs relevant to HSR. The structure of the HSR-relevant MPH programs varies across the seven SPHs.

**Table 3:** Structure of Masters in Public Health (MPH) curricula in schools of public health (SPHs) in East and Central Africa

**MPH program**	**Duration (years)**	**Average no. admitted per year**	**Average no. graduates per year**	**Mode of delivery-fulltime or distance**	**Credit hours**	**Duration of practicum (weeks)**	**Admission requirements**	**Assessment formative (F) and summative (S)**	**Full time staff in the school**	**Last curricula review prior to October 2011**
CPHMS, Ethiopia	2	80	65	Both	46	12 (Community-based education)	Multidisciplinary/at least 2 years’ relevant work experience andentrance exam	F = 50% S = 50%	80	2005–2006
KSPH, DRC	1	60	60	Fulltime/face to face	85	8 weeks	Postgraduate 3 years’ experience	F = 20% S = 70% PR = 10	38	2008–on-going
MakSPH, Uganda	2 –fulltime 3 –distance	80	15	Both	63	10 weeks field attachment	Multidisciplinary/at least 2 years’ work experience	F = 30% S = 70%	58	2009–2010
MUSOPH, Kenya	2	120	18	Fulltime/face to face	51	3 weeks practicum/course based	Multidisciplinary/at least 2 years’ relevant work experience	F = 40% S = 60%	22	2009–on-going
MUSPHSS, Tanzania	1	20	20	Fulltime/face to face	38.4	None	Public health/2 years’ work experience and entrance exam	F = 40% S = 60%	27	None
SPHUoN, Kenya	2	25	8	Fulltime/face to face	120	None	Multidisciplinary/2 years’ work experience	F = 30% S = 70%	18	2006–on-going
NURSPH, Rwanda	2	60	40	Fulltime/face to face	240	3 weeks field work	Multidisciplinary/2 years’ work experience in the health sector	F = 40% S = 60%	45	2008–2010

With respect to duration of the MPH program, MUSPHSS, Tanzania, and KSPH, DRC, host a 1-year program boasting graduation rates of 100% with an intake of 20 and 60 students per year, respectively. SPHs with 2-year programs have intakes ranging from 25 to 120 and graduation rates ranging from as low as 15% (MUSOPH, Kenya) and 19% (MaKSPH, Uganda), to 32% (SPHUoN, Kenya), to 66% (NURSPH, Rwanda) and 81% (CPHMS, Ethiopia). While it is clear that program duration influences graduation rates, this does not appear to be the case between intake and graduation rates. Only MakSPH, Uganda, and CPHMS, Ethiopia, offer an MPH program by distance learning. There is no standard definition of credit hours required to complete an MPH across the seven schools with required credit hours ranging from 38.4 (MUSPHSS, Tanzania) to 85 (KSPH, DRC) for the 1-year MPH and from 46 (CPHMS, Ethiopia) to 240 (NURSPH, Rwanda) for the 2-year MPH program.

Three other features that we reviewed on curriculum design were experiential learning (especially duration of field placements), admission criteria (focusing on multi-disciplinary background of trainees and years of relevant work experience), and the nature of program assessment (emphasis on continuous vis-à-vis summative assessment). Table 3 shows that four schools (CPHMS, Ethiopia; KSPH, DRC; MakSPH, Uganda; and MUSOPH, Kenya) offer non-thesis field placements, ranging from 3 to 12 weeks, designed to provide experiential learning. As of October 2011, MUSPHSS, Tanzania, and SPHUoN, Kenya, did not offer structured field-based learning experiences in their MPH training program. One key informant pointed out that research results are not making their way into policy and practice and suggested that SPHs need to perhaps include diverse and relevant methods for engagement with external stakeholders during curricula design and dissemination of research results in addition to building capacity of staff.

“*Some attempt is being made to train staff on research-related courses but again no emphasis on how to translate their finding to care, just gathering dust in the form of publications and theses. Publications are just used for career development by university lecturers.*” [KII, Ministry of Health, Kenya].

The admission criteria into the MPH programs are similar across the seven SPHs: a multi-disciplinary background with at least 2 years of relevant work experience. Interestingly, CPHMS, Ethiopia, and MUSPHSS, Tanzania, were the only SPHs that conduct entrance examinations to assess applicants.

On program assessment, the distribution of formative to summative course assessments was evenly split for CPHMS, Ethiopia, at 50%. This was followed by MUSOPH, Kenya, and MUSPHSS, Tanzania, with 40% formative to 60% summative. It is noteworthy to point out that these three SPHs were established or have adopted the problem-based learning and community-based education philosophy. Finally, for KSPH, DRC, MakSPH, Uganda, and SPHUoN, Kenya, were at 30% for formative to 70% summative, indicating a more traditional mode of assessment.

Adequacy and competency of human resources is a key attribute in the design of a competency-based curriculum [6]. The MPH programs are taught across departments, and, as Table 3 shows, the number of full-time staff in each SPH ranges from 18 (MUSOPH, Kenya) to 80 (CPHMS, Ethiopia) depending on the number of departments and schools that form the college of health sciences. While these absolute numbers cannot be used to infer adequacy and competency of staff and how they impact quality teaching and graduation rates, the variations in staffing suggest an important problem facing most of the SPHs – attraction and retention of competent staff. The two quotes below illustrate some of the factors relevant for attracting and retaining quality teaching staff.

“*The factors that have been found to be successful in attracting qualified staff include the fact that the teaching philosophy of the university is community-based education, limited bureaucracy in the university as compared to other universities, openness, and, to some extent, provision of housing.*” [KII, University Management, CPHMS, Ethiopia]

“*While a third of the staff are junior (37%; 16/43), with less than 10 years working experience, about 10 of the staff are about to retire or are employed on contract terms after their retirement. Although the school has a large number of well trained and experienced staff, the school is facing a challenge of keeping abreast with the new technologies for teaching competence curriculum in a large and expanding number of programs and student intake.*” [KII, Focal Person, MUSPHSS, Tanzania].

This information on staffing brings to the fore the issue of mentorship as an aspect of capacity building for teaching and innovation to training by using technology. These two are key aspects of a competency-based curriculum. All the staff who are about to retire at MUSPHSS, Tanzania, are doctoral degree holders and may worsen the staffing problem unless more staff are recruited.

Finally, it appears that most SPHs, except MUSPHSS, Tanzania, do review curricula (a key requirement for quality assurance), but these reviews are not regular for some and incomplete for others. For instance, three SPHs initiated and completed reviews in the years preceding the assessment in 2011. These include CPHMS, Ethiopia (2005 to 2006), NURSPH, Rwanda (2008 to 2010), and MakSPH, Uganda (2009 to 2010). Three other SPHs had initiated but not completed curricular reviews by December 2011, namely, SPHUoN, Kenya (2006), KSPH, DRC (2008), and MUSOPH, Kenya (2009). The quote below exemplifies the perspective of the slow-paced process of curriculum review.

“*Curriculum review is an exercise that usually involves different departments in the SPH, bringing all members of the department together takes a lot of effort and can drag on and on for a long time delaying the review process.*” [KII, University Staff, NURSPH, Rwanda].

### Perceived interest and competencies to teach HSR and implement HSR curricula

Table 4 shows three main results from staff at each school who responded to questions under the organizational capacity component of the self-assessment tool. The results are presented as proportions of respondents who agreed (score 4) or strongly agreed (score 5) as well as the mean scores of values from the Likert scale classified as strong, moderate, and weak under the methods section of this paper. The first set of results reveals staff perceptions on proportions of staff and students interested in HSR. In this study, this number of staff not only reflects the interest in HSR but also the capacity to teach and mentor students in HSR-relevant areas. The second set indicates staff perceptions of existing competencies (knowledge, quantitative, and qualitative skills) to teach HSR. Finally, Table 4 reveals perceived capacity of staff to design appropriate curriculum and access to learning and teaching resources (library). These results show the strengths, weaknesses, and challenges relevant to perceived capacity of faculty to teach HSR and implement HSR-relevant curricula in the seven SPHs.

**Table 4:** Perceived interests, capacities for designing, and competencies to teaching and mentoring health systems research (HSR) courses at the seven schools of public health (SPHs)

		**Perceived interest of staff and students in HSR**			**Perceived competencies for teaching HSR**			**Perceived capacity for designing evidence based courses**		
Name of SPH	Proportion of respondents based on number of staff in the school	Mean score and percentage of staff reporting adequate number of researchers in SPH interested in HSR % (mean score)	Mean score and percentage of staff reporting many graduate students at their SPH are interested in HSR % (mean score)	Mean score and percentage of staff reporting many undergraduate students at their SPH interested in HSR % (mean score)	Mean score and percentage of staff with strong quantitative skills interested in HSR % (mean score)	Mean score and percentage of staff with strong qualitative skills interested in HSR % (mean score)	Mean score and percentage of staff with adequate knowledge to teach HSR % (mean score)	Mean score and percentage of staff reporting their SPH offers courses relevant to HSR % (mean score)	Mean score and percentage of staff reporting courses provided draw upon appropriate literature and teaching materials % (mean score)	Mean score and percentage of staff reporting adequate library materials for teaching HSR % (mean score)
KSPH, DRC	92.1% (35/38)	85.7% (4.0)	22.9% (3.1)	48.6% (3.2)	77.1% (3.9)	51.5% (3.3)	82.9% (4.0)	65.7% (3.5)	28.6% (2.9)	14.3% (2.4)
MUSPHSS, Tanzania	37.2% (16/43)	62.5% (3.7)	25% (2.8)	62.5% (3.0)	62.5% (3.8)	62.5% (3.8)	68.75% (4.1)	68.75% (3.6)	50% (3.6)	6.25% (2.5)
NURSPH, Rwanda	21.1% (4/19)	0% (3.0)	25% (3.0)	25% (2.5)	100% (4.5)	0% (2.5)	25% (3.0)	100% (5.0)	25% (2.8)	0% (2.5)
MakSPH, Uganda	25.9% (15/58)	86.7% (4.0)	20% (3.1)	73.3% (3.9)	73.3% (4.2)	56.7% (3.9)	93.3% (4.1)	93.3% (4.1)	56.7% (3.7)	46.7% (3.2)
MUSOPH, Kenya	62.9% (22/35)	50% (3.9)	25% (3.3)	62.5% 3.9	62.5% 3.8	37.5% 3.4	62.5% 3.6	62.5% 3.7	62.5% 3.6	12.5% 2.5
SPHUoN, Kenya	27.8% (5/18)	60% (2.0)	60% (4.6)	0% (3.0)	0% (2.0)	0% (2.0)	60% (4.0)	60% (4.0)	0% (3.0)	0% (2.0)
CPHMS, Ethiopia	9.1% (26/285	61.5% (3.6)	53.9% (3.5)	65.4% (3.9)	73.1% (3.8)	57.7% (3.3)	57.7% (3.5)	73.1% (4.0)	46.2% (3.2)	57.7% (3.5)

SPHs differ in strength as well as share weaknesses across all the nine dimensions assessed. First, on perceived interest in HSR, all SPHs, except SPHUoN, Kenya (2.0), depicted a pattern of moderate (CPHMS, Ethiopia, NURSPH, Rwanda, and MUSPHSS, Tanzania) or strong (MakSPH, Uganda, KSPH, DRC, and MUSOPH, Kenya) interest among teaching staff. On the contrary, students were perceived by staff to have relatively less interest in HSR compared to teaching staff. Specifically, staff at MUSPHSS, Tanzania, perceived graduate students to have the least interest in HSR (mean score 2.8), while SPHUoN, Kenya, which registered the lowest mean score on interest among teaching staff, had the highest mean score on perceived interest among graduate students. The other four of the SPHs reflected a moderate average score ranging between 3.0 and 3.79. Interestingly, the three SPHs with strong perceived undergraduate student interest in HSR – MakSPH, Uganda (adopted after 2003), MUSOPH, Kenya, and CPHMS, Ethiopia – also had strong field placement programs founded on problem- and community-based learning principles. This suggests, in part, that such curriculum design spurs student interest through effective supervision/mentorship at undergraduate and graduate level.

Second, on perceived competencies (knowledge, quantitative, and qualitative skills) to teach HSR, all SPHs, except SPHUoN, Kenya, were perceived to have strong quantitative skills (63–77% agreed or strongly agreed). On the other hand, only two schools (MakSPH, Uganda, and MUSPHSS, Tanzania) registered relatively high mean scores (3.9) in qualitative skills required to support research and teaching of HSR.

Responses on capacity to design and teach HSR-relevant curricula were based on two questions in the self-assessment tool. Staff perceptions on whether staff have the knowledge to teach HSR-relevant courses and if the existing curricula have HSR-relevant content (staff able to design). Respondents across all the SPHs reported strengths in knowledge of HSR among staff with average scores in four SPHs (MakSPH, Uganda (4.1); KSPH, DRC (4.0); MUSPHSS, Tanzania (4.1); and SPHUoN, Kenya (4.0)) being perceived as strong and in three SPHs (NURSPH, Rwanda (3.0); MUSOPH, Kenya (3.6); and CPHMS, Ethiopia (3.5)) being perceived as moderate. Interestingly, on the perception as to whether the staff in the same SPHs had knowledge to design HSR-relevant courses (existing curricula have HSR-relevant content), there was a decrease in the proportion for KSPH, DRC (4.0 to 3.5) and MUSPHSS, Tanzania (4.1 to 3.6) but an increase in those holding the view that the same staff had capacity to design (NURSPH, Rwanda (3.0 to 5.0) and CPHMS, Ethiopia (3.5 to 4.0)). The other three MakSPH, Uganda, SPHUoN and MUSOPH (both in Kenya) had similar proportions holding the view on staff with knowledge to teach as well as design HSR-relevant courses. The variations between perceived capacity to teach and design the explanation is reflected in the poor interest in HSR among postgraduate students in these SPHs suggesting a need for mentorship programs.

Finally, Table 4 also reveals that staff in all SPHs were of the opinion that courses offered were relevant to HSR; four SPHs (NURSPH, Rwanda, MakSPH, Uganda, SPHUoN, Kenya, and CPHMS, Ethiopia) strongly held that opinion and three SPHs (KSPH, DRC, MUSPHSS, Tanzania, and MUSOPH, Kenya) held a moderate opinion. None of the staff in the seven schools held a strong opinion that resources for designing curricula, teaching, and learning HSR (library) were adequate. The mean scores ranged from 2.0 to 3.5 for library resources with KSPH, DRC, and NURSPH, Rwanda, being the least equipped with these resources.

### Opportunities for aligning HSR priorities to teaching programs

The final part of this paper explores the extent of opportunities for aligning HSR priorities to design or review teaching programs. In reviewing the information from the “quick and dirty” exercise that targeted external SPH partners, we found that none of the participating countries had a national agenda on HSR priorities, yet there appears to be general consensus among external stakeholders on priorities in HSR. When these identified priorities are juxtaposed against existing teaching programs offered at the seven universities as of October 2011, the analysis reflected in Table 5 reveals little convergence.

**Table 5:** Convergence of health system research (HSR) priorities and teaching programs

**Name of SPH**	**National HSR priorities from internal and external stakeholders**	**Convergence HSR priorities and teaching programs at each school**
KSPH, DRC	Health workforce, health financing, governance and supplies, commodities and technologies	No national HSR agenda but scope of priorities covers 6 building blocks; has 2 main programs
MUSPHSS, Tanzania	Service delivery, health financing, health workforce, health information, supplies, commodities and technologies	No national HSR agenda but scope of priorities covers 5 building blocks; has one relevant programs
NURSPH, Rwanda	Health financing, service delivery and supplies, commodities and technologies	No national HSR agenda but scope of priorities covers 3 building blocks; no relevant program
MakSPH, Uganda	Leadership and governance, service delivery, health financing, information systems and supplies, commodities and technologies, health workforce	No national HSR agenda but scope of priorities covers 6 building blocks; two relevant programs
MUSOPH, Kenya	Health financing, policy, service delivery, information systems and supplies, commodities and technologies, health workforce	No national HSR agenda but scope of priorities covers 6 building blocks; one relevant program
SPHUoN, Kenya	Health financing, health workforce, leadership and governance	No national HSR agenda but scope of priorities covers 3 building blocks; no relevant program
CPHMS, Ethiopia	Service delivery (5 areas)	No national HSR agenda and scope focused on 5 areas under one (service delivery) building block; one relevant program

Several opportunities exist to leverage the strengths of the SPHs in order to address the weaknesses in existing programs. Various stakeholders identified many national and regional opportunities relevant to curricula design and teaching of HSR, including developing eLearning platforms, sharing teaching and learning resources including case studies, developing a regional HSR curriculum, and establishing procedures for credit transfer across regional institutions. Perhaps these opportunities provide a platform for SPHs to consider when deliberating their capacity development plans.

## Discussion

To address inequalities in African health systems that may be linked to policymakers having limited access to and use of evidence from relevant bodies and professionals, in part requires competent health system researchers to generate this evidence. To impart the relevant knowledge, skills, and positive attitudes on HSR practitioners requires that appropriately trained and competent faculty deliver a well-resourced competency-based curriculum. This requirement is often overlooked as most previous capacity building initiatives have oftentimes focused on building competencies of HSR practitioners without establishing sustainable support for the training process [1-4,7,14].

The aim of this paper is to share and reflect on the nature of existing curricula for training public health students in HSR, the design of curricula, and the capacity to teach HSR at the seven universities in six countries across East and Central Africa. It also seeks to establish opportunities that SPHs could leverage to align national HSR priorities to the HSR curricula at national and regional level. Establishing the status of HSR-relevant curricula and institutional capacity of SPHs to design and deliver the curricula is a key phase of the journey towards developing appropriate curricula for transformative learning of the HSR professional and promoting national as well as regional interdependence in teaching of HSR as argued by various scholars [5,6].

Findings on context for curricula design and implementation should provide a clear justification for design of an HSR curricula at national and regional levels as well as a rationale for building capacity. Other scholars report that the countries share similar socio-economic and political challenges and their health systems continue to suffer familiar challenges, such as dilapidated infrastructure, weak referral systems, weak leadership, and poor management, which lead to inefficient and ineffective service provision and an imbalance between supply and demand for competent health professionals [4-6]. Since none of the six countries had either a national agenda for HSR or an HSR course, there is strong justification for engaging relevant stakeholders and designing a curriculum that fills this gap. Some agencies have argued for formal engagement of policymakers to use evidence from health research [19], and specific scholars recently described an integrated systems framework that can be used to understand the dynamic relationship between education and health [5]. We suggest a competency-based HSR curriculum and relevant capacity to teach that curriculum in order to integrate the two sub-systems. This was earlier defined as a curriculum that is student-centered, performance-oriented, and equips the learner with the knowledge, skills, and positive attitude to efficiently and effectively perform their current and anticipated tasks. A competency-based curriculum for HSR is essential in order to make training responsive to health systems needs [5]. Some known reasons for the slow and fragmented approach to adoption of educational reform include resistance to change educational philosophy during the formative years of an institution [4]. This observation is supported by two facts from these results. First, the oldest SPHs (MaKSPH, Uganda, and SPHUoN, Kenya) successfully transitioned to SPH later than those at recently established universities. Second, the two SPHs, which did not have traditional naming of departments, e.g., epidemiology and biostatistics, were purposively established on the educational philosophy of problem-based learning.

An additional finding from a key informant pointed out that research results are not making their way into policy and practice indicating that curricula need to perhaps include diverse and relevant methods for effective dissemination of research results to stakeholders. Evidence-based decision making is also essential, given that about 12% of the world’s population resides in sub-Saharan Africa, yet it is home to over 25% of the global disease burden and supported by only 3% of the world’s health workforce [4]. To ensure that HSR curricula are evidence-based, the consortium of seven SPHs in East and Central Africa collaborating under the Africa Hub on HSR will have to tap into portal resources such as those identified by the sub-Saharan African Medical Schools Study initiative funded by the Bill and Melinda Gates Foundation, which provides updates on the state of medical education in the region covering innovations and trends. However, at the moment, this initiative does not highlight HSR [4]. Since the completion of this study, CHEPSAA has developed relevant courses on complex health systems, health systems and policy research, and how to design curricula that SPHs should take into account in order to enhance HSR capacity [6]. The IUCEA is working on a regional qualification framework that would set minimum standards for select programs and allow credits to be transferred between universities. This would be very relevant for this group of SPHs that may seek to introduce a credit transfer system and may provide an opportunity to contribute to IUCEA and Association of Schools of Public Health in Africa discussions on minimum standards for HSR course content and structure, as well as enhancement of teaching capacities in the region. As other studies have recommended the strengthening of regional networks [4,6], the seven SPHs can leverage this supportive policy environment and existing networks and experiences in engaging these and other regional bodies to develop standards for HSR curricula across the region.

The results of this capacity assessment offer evidence of the differences among the seven SPHs in terms of the range and scope of their degree programs with regards to the concept of health systems, program durations, modes of delivery and assessment, graduation rates, and resources. These differences persist despite the fact that the SPHs have similar admission criteria, student backgrounds, and quality assurance policies to guide process of curricula development and teaching practices. These findings indicate that the existing curricula do not meet the requirements of a competency-based curriculum that emphasizes experiential learning using adult learning principles. Limited opportunities for appropriate placements into experiential learning settings further weaken the alignment of the curricula to both workplace environments and employee expectations. According to the WHO, the scope of a curriculum for a HSR degree program should cover all of the building blocks of the health system [1,2]. Equally important, failure to regularly review curricula suggests that the schools are overly bureaucratic and lethargic about engaging stakeholders in the process of continually improving the quality of their programs. Only three out of the seven SPHs had initiated and completed curricula review, and even in these cases the process was not very inclusive, because only a few key stakeholders were engaged as shown by the qualitative information on review at the departmental level.

There are major variations in the staffing levels across the seven SPHs. However, without additional data to establish teaching load among other factors, it would be misleading to infer any relative advantage or disadvantage based on absolute numbers. Moreover, most of the teaching staff do not have the prerequisite training in HSR. It would be important for each school to strive to meet the staff to student ratios prescribed by UNESCO [10] for university-level training, which is divided into strong (ratio of less than 20), medium (20 to 30), and weak (less than 30). The above shortcomings notwithstanding, only two of the SPHs implement distance learning/eLearning as an alternative delivery strategy and two had 100% graduation rates. Unfortunately, neither the alternative mode of delivery nor the high graduation rates can be associated with availability of full-time staff because of insufficient data. A formidable challenge all the universities face is declining trends in government funding used to maintain competitive salaries and provide staff development. In addition, governments are reducing investment in the development of learning facilities despite rapidly rising student enrollments [20,21]. This greatly reduces the number of staff with doctoral degrees, weakens research initiatives, and interferes with implementation of university and national policies, thus further weakening the link between university-level training by SPHs and global public health objectives.

The capacity of staff to teach and mentor HSR-relevant courses was generally weak across the seven SPHs since most faculty had training in the traditional research methods and lack a health systems-specific background and/or training. There are opportunities for sharing resources across the seven SPHs as well as to develop dual career pathways to mentor junior faculty to take up HSR training. This conclusion has been reached by other studies examining data across different universities throughout Africa [4,6].

As results showed, most of the SPHs were perceived to have strong policies and procedures to design curricula drawing on a wide range of resources. It is also true that there exists basic infrastructure to design online courses, but only one SPH offered HSR-relevant curricula online. There is a strong demand for health system professionals but the supply is limited and to address the imbalance in the system new curricula should respond to both the needs of the community and priorities of the health sector. Frenk et al. point out that evaluating appropriateness of current curricula to the HSR priorities and needs of the community requires the education system to diversify training strategies so as to produce health professionals with the appropriate skill mix and competencies [5]. One way of doing this is to engage key stakeholders to determine the priority health system needs and then develop an inclusive agenda. This was partially done in this study and revealed convergence between health system priorities and existing curricula relevant to HSR. Another approach is to design appropriate field placements during training which ensure experiential learning and can also help to align the curricula to workplace competencies in health systems management. Five of the SPHs had some form of field practicum; however, the data do not permit an in-depth analysis of the structure and content of these practicums to judge their effectiveness.

In the final section, we present a set of recommendations that can be used to improve the institutional capacity to design and teach HSR teaching curricula within and across the SPHs as well as at regional levels in order to improve health system responsiveness to the needs of communities. Overall, the study adds value to the existing body of knowledge in three ways. First, having infrastructure and a supportive environment to design and teach HSR-relevant curricula alone is not sufficient. Instructional factors, such as conceptual clarity, interest, knowledge, and skills, are important. Second, competency-based approaches have not taken root in most SPHs and in the few where limited educational changes in philosophy have occurred, the implementation is largely partial and/or piecemeal. Finally, although opportunities exist for designing quality curricula and offering learner-centered training in HSR in SPHs across East and Central Africa, most SPHs have not leveraged their strengths to build institutional capacity using national and regional networks.

## Conclusions and recommendations

Overall, although SPHs share similar institutional contexts and some capacity to design competency-based curricula, such as problem-based learning, multi-disciplinary focus, and experiential learning, and have the infrastructure and/or potential to develop it through the rich range of networks and partnerships, the seven SPHs are mostly ill-prepared for transformative learning for the HSR professional. This is exemplified by the lack of an HSR-specific curriculum at any of the SPHs at the time of the survey and the lukewarm interest in HSR research and training amongst graduate students compared to enthusiastic external stakeholders giving clear but varied health system priorities. These findings are akin to what other scholars surveying various schools of medicine in Africa have observed – a slow-paced transition to systems-based learning, piecemeal adoption of existing technology to transform teaching and learning, and working in silos rather than building strategic networks to develop and sustain institutional capacities for interdependence in teaching HSR [4-6].

### Program-level recommendations

These are based on the evidence of a diverse context, lack of HSR curricula, varied capacities to design and teach, and untapped opportunities at national and regional level; the SPHs proposed these interventions.

(i) Develop school-specific, national and regional capacity building plans to strengthen instructional capacity of staff and institutional capacity of SPHs to design and teach HSR.

(ii) To jointly develop and implement a standardized regional HSR curriculum that is competency-based and emphasizes a systems approach as a pilot.

(iii) Participating countries should consider developing an HSR eLearning platform for the regional course.

(iv) Based on the diverse strengths, the SPHs should promote sharing of experiences and practices with respect to teaching HSR, and, where gaps exist, develop an inventory of shared HSR teaching materials and through teacher and student exchange.

(v) Depending on institutional practices at each SPH, existing curricula should be reviewed with the aim of integrating the HSR syllabus, especially at undergraduate level. This is likely to promote interest and mentorship as part of teaching across all SPHs.

### Policy-level recommendations

At the policy level, schools should review relevant procedures and encourage their respective University Senates to support capacity-building initiatives for designing competency-based philosophy into the HSR degree programs. These changes would not only improve the efficiency and effectiveness of these training programs but also promote the culture of evidence-based policy-making.

As part of curricula design, there is a need to explore the potential for credit transfer among the seven SPHs. This may require support from regional bodies such as the IUCEA among other regional networks engaged in standardization and harmonization and quality assurance. An effective credit transfer system would increase demand and improve mobility between universities for the HSR programs across the six countries and the region.

To further stimulate interest in teaching HSR, the schools should explore the potential for a regional mentoring program for junior faculty as well as an eLearning in-service course to expand access but also stimulate more interest in HSR among health professionals. These two strategies will also strengthen the linkages among academicians/teachers, researchers, and policymakers, a point stressed by some of the KII respondents from the Ministries of Health.

Finally, at the institutional level, all SPHs need to review their policies on collaborations and alliances with industry to support experiential learning. Notwithstanding, policymakers should have clear implementation plans for operationalization of these policies. This will assure that students receive early exposure to real workplace environments and transformative learning that leads to improved outcomes and performance among health professionals. In addition, institutions should work with key stakeholders to consolidate and streamline funding streams. This could lead to various options for regional scholarships for HSR. An institutionalized tracking system for the graduates in HSR and related degree programs is also urgent.

### Further research

To better inform the process of curricula design and review, we recommend establishing and institutionalizing a monitoring and evaluation system for HSR graduates of the MPH programs to determine gaps in HSR training at both the undergraduate and graduate levels. Finally, a follow-up study around the systems-based competency-driven framework to unearth specific instructional gaps across the seven institutions in capacity building for HSR is recommended.

## Abbreviations

CHEPSAA: Consortium for Health Policy and Systems Analysis in Africa; CPHMS: College of Public Health and Medical Sciences; DRC: Democratic Republic of the Congo; FP: Focal Person; HEALTH: Higher Education Alliance for Leadership Through Health; HSR: Health Systems Research; IDRC: International Development Research Centre; IUCEA: Inter-University Council of East Africa; KII: Key informant interview; KSPH: Kinshasa School of Public Health; MakSPH: Makerere University College of Health Sciences; MPH: Master of Public Health; MUSPHSS: Muhimbili School of Public Health and Social Sciences; MUSoPH: Moi University, School of Public Health; NURSPH: National University of Rwanda School of Public Health; SPH: School of Public Health; SPHUoN: University of Nairobi School of Public Health.

## Competing interests

The authors do not have any competing interests to declare.

## Authors’ contributions

All four authors were involved in the design and implementation of the assessment tool. MN wrote drafts of the manuscript and coordinated all inputs. BT, LR, and AM provided substantial input, as well as reviewing the draft manuscript. All four authors read and approved the final manuscript.
